# Urinary microbiota in bladder cancer: insights into pathogenesis, diagnosis, and therapeutic potential

**DOI:** 10.3389/fcimb.2026.1890640

**Published:** 2026-08-20

**Authors:** Zhaoyang Sheng, Jinpeng Zhu, Jianming Sun, ShuXiong Zeng, Jun Ding, ZhenSheng Zhang

**Affiliations:** 1 Department of Urology, Shanghai Changhai Hospital, Naval Medical University, Shanghai, China; 2 Department of Urology, The 904th Hospital, Joint Logistics Support Force, Wuxi, China

**Keywords:** BCG, bladder cancer, mechanism, microbiota, treatment

## Abstract

Bladder cancer is a prevalent urinary malignancy with high global incidence and mortality. Despite advances in diagnosis and treatment, challenges persist, particularly due to high recurrence rates and unpredictable progression. Recent evidence highlights the role of urinary tract microbiota in BC initiation, progression, and recurrence. The urinary tract, once thought sterile, is now recognized as a complex microbial environment influencing local immunity and carcinogenesis. Differences in microbiota composition between cancer patients and controls, as well as variations by gender, age, and lifestyle factors like smoking, have been observed. The microbiota may impact tumor staging, grading, recurrence, and treatment responses, particularly to Bacillus Calmette-Guérin therapy. Mechanisms include immune modulation, inflammation, and epithelial barrier disruption. This review synthesizes current research on urinary microbiota’s role in BC, emphasizing its potential as a biomarker and therapeutic target. Large-scale studies are needed to better understand these associations and develop microbiota-based strategies for improved BC management.

## 1 Introduction

Bladder cancer (BC) is one of the most common malignancies of the urinary tract, accounting for an estimated 614,298 new cases and 220,596 deaths worldwide in 2022 (Bray et al., 2024). The incidence of BC has shown an upward trend in recent years (van Hoogstraten et al., 2023). At diagnosis, about 75% of patients present with non-muscle invasive BC (NMIBC), which can often be managed effectively through transurethral resection. However, BC is characterized by a high recurrence rate, with 50-70% of NMIBC patients experiencing recurrence within five years, and 10-30% progressing to muscle-invasive BC (MIBC) (Sylvester et al., 2016). This high incidence and recurrence impose a significant social and economic burden. Unfortunately, the etiology and pathogenesis of BC remain largely unclear.

In recent years, new evidence has challenged the traditional belief that urine in healthy individuals is sterile (Wolfe and Brubaker, 2015). Studies now suggest that the urinary microbiota plays a significant role in modulating local immunity and inflammatory responses within the urinary tract (Jones-Freeman et al., 2021). This interaction may profoundly affect the progression of inflammation-associated diseases, including interstitial cystitis, urgency incontinence, and neurogenic bladder dysfunction (Fouts et al., 2012; Pearce et al., 2014; Abernethy and Tsuei, 2021). Chronic microbial infections have been estimated to contribute to 20-30% of cancers (Baffy, 2020). Recently, increasing research has linked urinary tract microbiota to the onset and development of BC, suggesting its potential as a biomarker and therapeutic target (Bi et al., 2019a; Karam et al., 2022; Yacouba et al., 2022; Heidar et al., 2023). However, several limitations exist in the literature decreasing the reliability of the current reports (Stamatakos et al., 2024). For example, factors influencing the composition and diversity of urinary microbiota include age, gender, smoking habits, sampling methods, and antibiotic use, all of which may impact the results and interpretations of studies on urinary microbiota and its role in BC.

At present, the relationship between the urinary microbiota and BC remains poorly understood, with inconsistent findings across studies. Further research is needed to elucidate the specific mechanisms underlying this association and to establish consensus conclusions. Additionally, the potential of urinary microbiota as a tool for the prevention and management of BC remains largely unexplored, highlighting a significant gap in current research. This review explores the characteristics, influencing factors, mechanisms, prognosis, and therapeutic implications of the urinary microbiota in BC, aiming to provide novel strategies for early diagnosis, prevention, and management.

## 2 Factors affecting urinary microbiota

### 2.1 Gender differences

The incidence of BC in men is approximately three to four times higher than in women, though female patients tend to have a worse prognosis (Ferlay et al., 2015). Studies suggest that urinary tract infections (UTIs) may increase BC risk, and women, being more susceptible to UTIs, might exhibit different microbiota profiles compared to men (Stone, 2014). For instance, Fouts et al. (2012) reported that the order Lactobacillales was more abundant in women, whereas the genus *Corynebacterium* was more common in men. *Lactobacillus*, a Gram-positive genus frequently abundant in the urinary microbiota of women, may contribute to colonization resistance against uropathogens and protection from urinary tract infection (Kim and Park, 2018; Pohl et al., 2020). In contrast, *Corynebacterium*, which can hydrolyze lipids and release antimicrobial free fatty acids, remains controversial regarding its role in the urinary microbiota (Chen et al., 2018).

**Figure 1:**
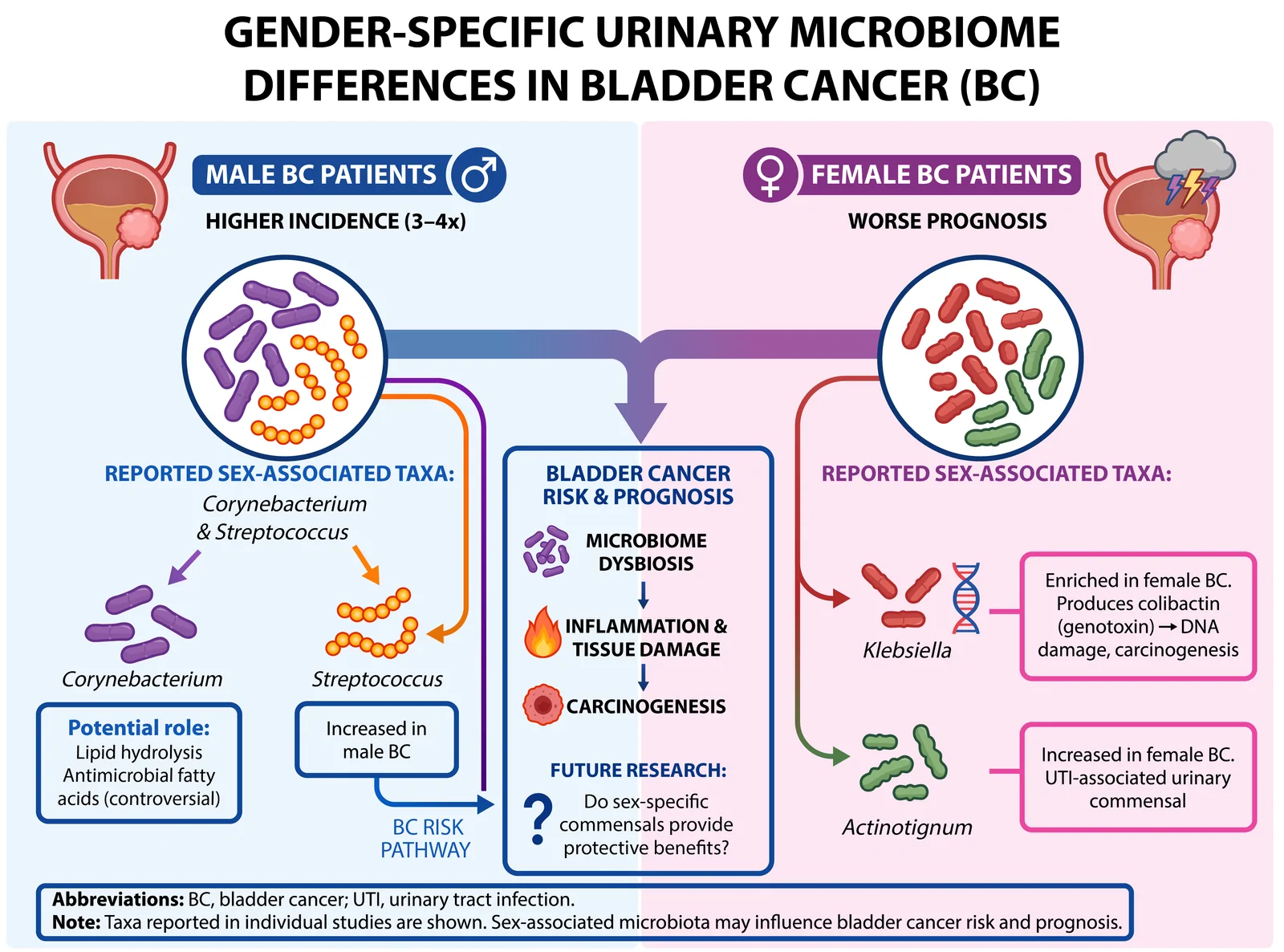
Gender-specific urinary microbiome differences in bladder cancer (BC). The schematic summarizes sex-associated urinary microbial taxa reported in individual bladder cancer cohorts. *Corynebacterium* and *Streptococcus* are shown as male-associated taxa, whereas *Klebsiella* and *Actinotignum* are shown as female-associated taxa. The central pathway illustrates a proposed link between microbial dysbiosis, inflammation, tissue damage, and bladder carcinogenesis. These associations are exploratory and should not be interpreted as established causal relationships.

In a study involving 49 BC patients and 59 healthy controls, Pederzoli et al. (2020) identified an enrichment of *Klebsiella* in female BC patients. Interestingly, *Klebsiella*, known to produce the genotoxin colibactin, has been associated with DNA damage, genomic instability, and carcinogenesis (Kaur et al., 2018; Quaglio et al., 2022). Additionally, *Klebsiella* has been linked to colorectal and gastric cancers, suggesting a potential role in BC pathogenesis (Alomair et al., 2018; Gantuya et al., 2019). A meta-analysis conducted by Yacouba et al. (2022) revealed a gender-specific difference in the bladder microbiome among BC patients: an increase in *Actinotignum* in female patients and an increase in *Streptococcus* in male patients. *Actinotignum*, classified under the class Actinobacteria in the phylum Actinobacteria, is a known urinary commensal associated with urinary tract infections (Lotte et al., 2016). Currently, data describing gender-specific characteristics of the bladder microbiome are limited, and further microbiome studies are needed to determine whether sex-specific commensals provide protective benefits to the host. The role of the urinary microbiota in the pathogenesis and progression of BC in both males and females remains to be further elucidated. These typical gender-related microbial characteristics are clearly illustrated in **Figure 1**.

### 2.2 Age-related changes

The risk of BC increases with age, with the majority of new cases diagnosed in individuals over 65 (Sanli et al., 2017). Age-related changes in urinary microbiota composition have been documented. For instance, Viljemka et al. (Hrbáček et al., 2023) identified *Jonquetella* as a characteristic bacterium in the urinary microbiota of individuals aged 70 and above, though the relationship between these microbial shifts and cancer risk remains unclear. Another study on age-related changes in the male urinary microbiota reported a decrease in total bacterial load but an increase in bacterial diversity with age (Lewis et al., 2013). Curtiss et al. (2018) observed that premenopausal women had a higher abundance of *Lactobacillus* compared to postmenopausal women, while *Mobiluncus* showed the opposite trend. This discrepancy may be attributed to decreased estrogen levels after menopause, leading to vulvovaginal atrophy, weakened defense against pathogenic bacteria, and an increased risk of urinary tract infections (Heidar et al., 2023). Additionally, older men are more likely to experience incomplete bladder emptying due to prostatic hyperplasia, resulting in urinary retention and potential microbial colonization within the bladder (Luthje et al., 2014). These findings underscore the importance of considering age as a factor influencing urinary microbiota composition in BC research.

### 2.3 Regulatory effect of smoking

Smoking is a well-established risk factor for BC, with epidemiological evidence indicating that nearly one-third of BC patients have a history of smoking (Freedman et al., 2011). Results from a multicenter case-control study indicate that smokers have a higher risk of developing BC compared to non-smokers (Rink et al., 2015). Studies on urinary microbiota show conflicting findings, with Moynihan et al. (2019) reporting no impact of smoking on the urinary microbiota of male hematuria patients, whereas Ma et al. (2021) observed that smokers exhibited a higher abundance of Bacteroidaceae, Erysipelotrichales, Lachnospiraceae, and *Bacteroides* in the urinary tract compared to non-smokers. Alpha diversity was increased in smokers with BC, and principal component analysis (PCA) revealed significant differences in microbial composition between smokers and non-smokers. Whether smoking affects the microbiome in the bladder remains unknown, the observed alterations in the urinary microbiota could be attributed to aromatic amines and polycyclic aromatic hydrocarbons in cigarette smoke, which may lead to DNA adduction and mutagenicity in the urothelium (Besaratinia and Tommasi, 2013), contributing to BC risk. More research is needed to elucidate the specific mechanisms by which smoking alters the urinary microbial composition.

### 2.4 Influence of specimen collection methods

The choice of urine collection method can significantly impact the composition of urinary microbiota (Luthje et al., 2014). Urine specimens may be obtained by voided collection, including first-catch or midstream urine, by intermittent or indwelling catheterization, by cystoscopic collection, or by suprapubic aspiration. Each collection method samples a somewhat different anatomical microbial compartment and may therefore yield distinct microbial profiles. Suprapubic aspiration obtains urine directly from the bladder and can minimize contamination from the urethral, genital, or skin microbiota; however, it is the most invasive urine collection method and is therefore rarely used in routine urinary microbiome studies. Catheterized or cystoscopically collected urine is less affected by microbial contributions from the distal urethra and genital tract and may more closely approximate the bladder luminal microbiota. However, these approaches are invasive and are generally more appropriate when urinary instrumentation is clinically indicated. Many studies utilize midstream urine, while others rely on catheterization or cystoscopic sampling. A recent meta-analysis indicated that catheterized urine samples typically exhibit reduced abundance of several genera, potentially due to reduced contamination from the urethra, vagina, and skin (Bukavina et al., 2023). Catheterized samples often show lower levels of *Lactobacillus*, *Corynebacterium*, and *Cutibacterium*, known skin contaminants (Byrd et al., 2018).

Among non-invasive approaches, first-catch urine is more likely to contain microorganisms derived from the urethra, periurethral region, and genital tract, whereas midstream clean-catch urine is intended to reduce these distal microbial contributions. Genital or vaginal cleansing before sampling may further affect the relative abundance of skin- and genital-associated taxa. Therefore, the same preparation and collection protocol should be applied consistently to all participants and reported in sufficient detail. Another study reported that catheterized urine from BC patients showed increased levels of *Veillonella*, whereas voided urine samples had higher abundances of *Acinetobacter*, *Actinomyces*, and *Aeromonas *(Yacouba et al., 2022). Oresta et al. (2021) observed that the microbial profiles in male BC patients differed based on collection method, highlighting the potential limitations of voided urine in accurately representing bladder microbiota. Studies by Bajic et al. (2020), Kang et al. (2025) and Hrbáček et al. (2021) also confirmed that the microbial composition of catheterized urine samples differs from that of voided urine samples, indicating that voided urine may not adequately represent the bladder microbiota. However, Ozer et al. (2021) found no statistically significant difference in microbiota composition between initial voided and midstream urine, suggesting that both may be suitable for microbiota analysis when standardized collection procedures are used. Nevertheless, this finding does not exclude possible effects of sex, genital preparation, disease status, or other cohort-specific factors.

The optimal collection method should therefore be selected according to the study objective. Catheterized urine may be preferred when bladder-specific microbial characterization is required and invasive collection is clinically justified. In contrast, standardized midstream clean-catch urine provides a practical option for non-invasive diagnostic studies, population-based screening, and repeated longitudinal sampling. Future BC microbiome studies should clearly report the sampled void fraction, genital or vaginal preparation procedure, collection time, recent antibiotic exposure, urinary instrumentation, storage conditions, and laboratory-processing workflow. Samples obtained using different collection methods should be analyzed separately or adjusted for collection method in the statistical analysis. These methodological differences underscore the need for standardized urine collection protocols in urinary microbiome research.

### 2.5 Comparison of microbiota detected in urine and bladder tissue samples

Most studies on bladder microbiota rely on urine samples, whereas relatively few have examined bladder tissue, and the available studies have yielded heterogeneous findings. Liu et al. (2019) reported that Proteobacteria dominated the BC tissue microbiota, with significant reductions in both alpha and beta diversity. Conversely, Mansour et al. (2020) found that Firmicutes was the most abundant phylum in BC tissue, with no significant differences in microbial diversity compared to controls. These discrepancies may reflect differences in patient populations, tumor characteristics, sampling location, prior treatment or urinary instrumentation, sample-processing procedures, sequencing methods, and contamination control in low-biomass specimens.

In an intervention-naive paired-sample study, Pederzoli et al. (2020) reported that urine and bladder tissue shared up to 80% of bacterial families. However, this taxonomic overlap does not imply that the two compartments are spatially or ecologically equivalent. Urine mainly reflects free-floating microorganisms and microbial products within the urinary lumen, whereas tissue samples are more likely to capture adherent, mucosa-associated, and tumor-associated communities. Tumor-related alterations in urothelial architecture, inflammation, necrosis, oxygen availability, and local immune activity may further contribute to spatial discordance. Therefore, urine and bladder tissue should be considered complementary rather than interchangeable microbial compartments. Although urine-derived microbial profiles and bladder tissue-associated microbiota are not fully concordant, microbial features detected in urine may have greater clinical utility for monitoring BC progression because urine sampling is less invasive and more feasible than tissue biopsy.

## 3 Urinary microbiota associated with BC

### 3.1 Urinary microbiota composition in BC patients

Comparing the microbial composition between BC patients and non-cancer controls provides insights into the microbiota’s role in tumorigenesis. Research indicates significant differences in urinary microbiota between these two groups (Kustrimovic et al., 2024). A prospective multicenter cohort study by Bukavina et al. (2023) analyzed urinary microbiota from 129 patients with BC and 60 non-cancer controls across four countries. They reported elevated levels of *Sphingomonas*, *Acinetobacter*, *Micrococcus*, and *Pseudomonas* in BC patients. Similarly, Hrbáček et al. (2023) reported lower microbial richness and diversity in patients with BC than in controls, with a higher abundance of *Veillonella*, *Varibaculum*, and *Methylobacterium-Methylorubrum* in the cancer group, whereas *Pasteurella*, *Corynebacterium*, and *Acinetobacter* were more prevalent in controls. In one study, Deinococcus-Thermus phylum was found to be more abundant in BC (Hussein et al., 2021), while in another study, it was found to be more abundant in the control group (Liu et al., 2019).

At the phylum level, Bučević Popović et al. (2018) found that healthy controls harbored urine microbiota predominantly composed of Firmicutes, Actinobacteria, Bacteroidetes, and Proteobacteria, with comparable overall microbial diversity relative to BC patients. However, patients with BC displayed significantly elevated relative abundances of *Fusobacterium*, *Actinobaculum*, *Facklamia*, and *Campylobacter*. In some studies, elevated relative abundances of Actinobacteria and Proteobacteria have been detected among BC patients (Bi et al., 2019b; Liu et al., 2019; Hussein et al., 2021)., while Firmicutes were more abundant in controls (Liu et al., 2019; Hussein et al., 2021). Hussein et al. (2021) found no significant difference in the alpha diversity of the microbiota between BC patients and control groups, although there was a significant difference in beta diversity at the genus level. In contrast, Oresta et al. (2021) observed that the alpha diversity, specifically the evenness index, was significantly higher in BC patients, while beta diversity showed no significant difference. On the other hand, Moynihan et al. (2019) and Bučević Popović et al. (2018) reported no significant differences in either alpha or beta diversity.

A comprehensive summary of recent studies comparing microbiota detected in urine and bladder tissue samples from patients with BC and controls is presented in **Table 1**. These studies indicate variation in microbial taxa such as *Streptococcus*, *Acinetobacter*, and Fusobacteria across geographical locations and patient populations, reflecting the complexity and heterogeneity of BC-associated microbiota. Discrepancies in findings regarding which taxa are associated with BC may be attributed to differences in sample size, low-biomass sample handling, collection methods, demographic factors such as age and sex, antibiotic exposure, sequencing platforms, 16S rRNA target regions, and bioinformatic pipelines. Therefore, current findings should be interpreted as associative observations rather than causal mechanisms or clinically validated biomarkers, and large-scale, standardized, multicenter prospective studies are warranted to clarify the relationship between urinary tract microbiota and BC.

**Table 1:** Summary of studies analyzing microbiota in urine and bladder tissue samples from patients with bladder cancer and controls.

References	Country	Total N (Case/Control)	Sex distribution	Sample	Method	Key microbial findings (relative to controls)
Xu et al. (2014)	USA	14 (8/6)	NR	Urine	16sRNA	↑ *Streptococcus*
Wu et al. (2018)	China	49 (31/18)	Patients: 31 M/0 F; Controls: 18 M/0 F	Urine	16sRNA	↑ *Acinetobacter*, *Anaerococcus*, *Sphingobacterium*; ↓ *Serratia*, *Proteus*, *Roseomonas*
Bučević Popović et al. (2018)	Croatia	23(12/11)	Patients: 12 M/0 F; Controls: 11 M/0 F	Urine	16sRNA	↑ Fusobacteria
Mai et al. (2019)	China	24(24/0)	Patients: 18 M/6 F; Controls: NA	Urine	16sRNA	↑ *Acinetobacter*
Bi et al. (2019c)	China	55 (29/26)	Overall: 35 M/20 F; group-specific sex distribution NR	Urine	16sRNA	↑ *Actinomyces europaeus*
Liu et al. (2019)	China	34 (22/12)	34 M/0 F	Tissue	16sRNA	↑ *Cupriavidus, Acinetobacter, Escherichia-Shigella, Ralstonia* etc.; ↓ *Lactobacillus, Prevotella_9*, Ruminococcaceae
Moynihan et al. (2019)	USA	41 (8/33)	41 M/0 F	Urine	16sRNA	Most common genera: *Turicibacter, Lactobacillus, Bacteroides* (no case-control comparison)
Chipollini et al. (2020)	USA	35(25/10)	NR	Urine	16sRNA	↑*Bacteroides, Faecalibacterium*
Mansour et al. (2020)	Hungary	10 (10/0), 10 urine and 14 tissue samples	Patients: 5 M/5 F; Controls: NA	Urine and Tissue	16sRNA	Over-representation of *Akkermansia, Bacteroides, Clostridium (s.s.), Enterobacter, Klebsiella* in tissue vs. urine
Pederzoli et al. (2020)	Italy	108(49/59) 29	Patients: 36/13; Controls: 34/25 Patients: 21/8	Urine Tissue	16sRNA	Urine: *Klebsiella* (female BCa> healthy); Tissue: *Burkholderia* (neoplastic > non-neoplastic, both sexes)
Hussein et al. (2021)	Egypt	53 (43/10)	Patients: 36 M/7 F; Controls: 5 M/5 F	Urine	16sRNA	Beta-diversity differed; ↑ *Actinomyces, Achromobacter, Brevibacterium, Brucella*. MIBC vs NMIBC: ↑ *Hemophilus, Veillonella* (MIBC)*; ↑ Cupriavidus* (NMIBC)
Oresta et al. (2021)	Italy	61 (51/10)	Patients: 51 M/0 F; Controls: 10 M/0 F	Urine	16sRNA	Phylum level top 4: Firmicutes, Actinobacteria, Bacteroidetes, Proteobacteria
Chorbińska et al. (2023)	Poland	25 (18/7)	Patients: 14 M/4 F; Controls: 5 M/2 F	Urine	16sRNA	BCG-treated: ↑ *Lactobacillus*; female: ↑ *Howardella* and *Streptococcus anginosus*
Hrbáček et al. (2023)	Czech Republic	63 (34/29)	63 M/0 F	Urine	16sRNA	↑ *Veillonella, Varibaculum, Methylobacterium-Methylorubrum*
Heidrich et al. (2024)	Brazil	73 (32/41)	Patients: 32 M/0 F; Controls: 41 M/0 F	Urine	16sRNA	↑*Lactobacillus, Streptococcus, Cutibacterium* in patients without persistent disease
Tao et al. (2026)	China	155(111/44)	NR	Urine	16sRNA	↑*Anaerococcus, Fenollaria, inegoldia;*↓ *Bacteroides, uribaculaceae, herichia-Shigella*
Sheng et al. (2025)	China	300(104/196)	Patients: 89 M/15 F; Controls: 115 M/81 F	Urine	16sRNA	↑ *Sphingomonas*, *Anaerococcus*, *Acinetobacter*, *Stenotrophomonas*, *Aeromonas*, *Novosphingobium*;↓ *Lactobacillus*, *Gardnerella*
Jia et al. (2026)	China	27(15/12)	Patients: 14 M/1 F	Tissue	16sRNA	↑*Bacillus*, Bacillaceae, *Thermoomonas*, Enterococcaceae, *Enterococcus*

BC, bladder cancer; NMIBC, non-muscle-invasive bladder cancer; MIBC, muscle-invasive bladder cancer; BCG, Bacillus Calmette–Guérin; M, male; F, female; NR, not reported; NA, not applicable. ↑, significantly increased; ↓, significantly decreased.

### 3.2 Potential mechanisms by which microbial dysbiosis contributes to bladder carcinogenesis

Dysbiosis, or an imbalance in the microbiota, has been increasingly implicated in BC development and progression. However, most available human studies are cross-sectional and cannot determine whether microbial alterations precede tumor development or arise secondarily within the tumor microenvironment. Therefore, enrichment of a specific microbial taxon in BC should not, by itself, be interpreted as evidence of causality. This microbial imbalance has been associated with associated with a higher abundance of opportunistic pathogens and may contribute to carcinogenesis through various mechanisms, including disruption of the epithelial barrier, chronic inflammation, induction of genetic mutations, and modulation of tumor-related signaling pathways (Nicolaro et al., 2020; Wong-Rolle et al., 2021). One key mechanism may involve extracellular matrix (ECM) degradation by bacterial enzymes such as collagenase, hyaluronidase, and elastase, which may impair epithelial integrity and contribute to chronic inflammation—an established risk factor for BC (Alfano et al., 2016; Huang et al., 2021). Chronic inflammation, in turn, promotes the recruitment of inflammatory cells and the release of pro-inflammatory molecules, activating signaling pathways involved in tumor initiation and progression (Nwabo Kamdje et al., 2023). Furthermore, microbial products may contribute to genotoxic stress directly, when specific genotoxins or metabolites have been experimentally validated, or indirectly through reactive oxygen species (ROS) and reactive nitrogen species (RNS) generated during chronic inflammation. These processes may induce epithelial injury and genomic instability (Singh et al., 2019; Nicolaro et al., 2020).

The relationship between microbial dysbiosis and BC may also be bidirectional. Tumor-associated urothelial disruption, tissue necrosis, altered oxygen and nutrient availability, urinary retention, and impaired local immune surveillance may create ecological niches that favor secondary colonization by opportunistic microorganisms. Thus, some microbial enrichments may reflect adaptation to an already altered tumor microenvironment rather than a primary role in carcinogenesis.

Certain pathogens and chronic infections, rather than bacterial infections alone, have been linked to an increased risk of BC. A well-established example is *Schistosoma haematobium*, a parasitic flatworm associated with urogenital schistosomiasis, which promotes bladder carcinogenesis through egg deposition in the bladder wall, persistent granulomatous inflammation, oxidative stress, tissue remodeling, and repeated urothelial injury (Mostafa et al., 1999). Parasite-derived antigens and infection-associated inflammatory signals may be sensed by host pattern-recognition receptors; for example, *S. haematobium* infection has been associated with TLR2-mediated pro-inflammatory responses, while TLR4-related responses may also participate in pathogen-associated inflammatory signaling (Kawai and Akira, 2010; Meurs et al., 2011). Importantly, TLR signaling in BC should be interpreted as context dependent rather than uniformly activated or suppressed. In bladder tumors, especially non-muscle-invasive tumors, TLR expression has been reported to be decreased but still detectable, which may alter microbial recognition, local immune surveillance, and inflammatory regulation (LaRue et al., 2013).

Additionally, Chronic inflammation, cellular regeneration, and sustained epithelial injury may increase genomic instability, potentially contributing to BC development (Elinav et al., 2013). Studies have identified systemic inflammatory markers, such as the neutrophil-to-lymphocyte ratio (NLR), as predictive indicators of disease recurrence and progression in BC patients (Liu et al., 2018). Additionally, microbiota have been shown to influence BC prognosis through immune modulation. For instance, increased levels of FoxP3+ regulatory T cells (Tregs), Ki-67 expression, and microbial indices in non-muscle-invasive BC (NMIBC) have been correlated with poor prognosis, as higher Ki-67 levels are associated with more aggressive disease (Qiu et al., 2022). However, these associations may reflect complex interactions among tumor biology, host immunity, and the local microbial environment and do not establish a direct microbial cause of disease progression.

Microbial involvement also extends to the modulation of immunotherapy efficacy. Wang et al. (2024) demonstrated that the gut bacterium *Blautia coccoides* and its metabolic products enhance the efficacy of BC immunotherapy by promoting CD8+ T cell infiltration. Conversely, the microbiota may promote immune evasion through upregulation of the PD-L1/PD1 axis, a mechanism linked to cancer recurrence and progression in NMIBC (Chen et al., 2022; Zhang et al., 2023). However, these associations may reflect complex interactions among tumor biology, host immunity, and the local microbial environment and do not establish a direct microbial cause of disease progression.

**Figure 2:**
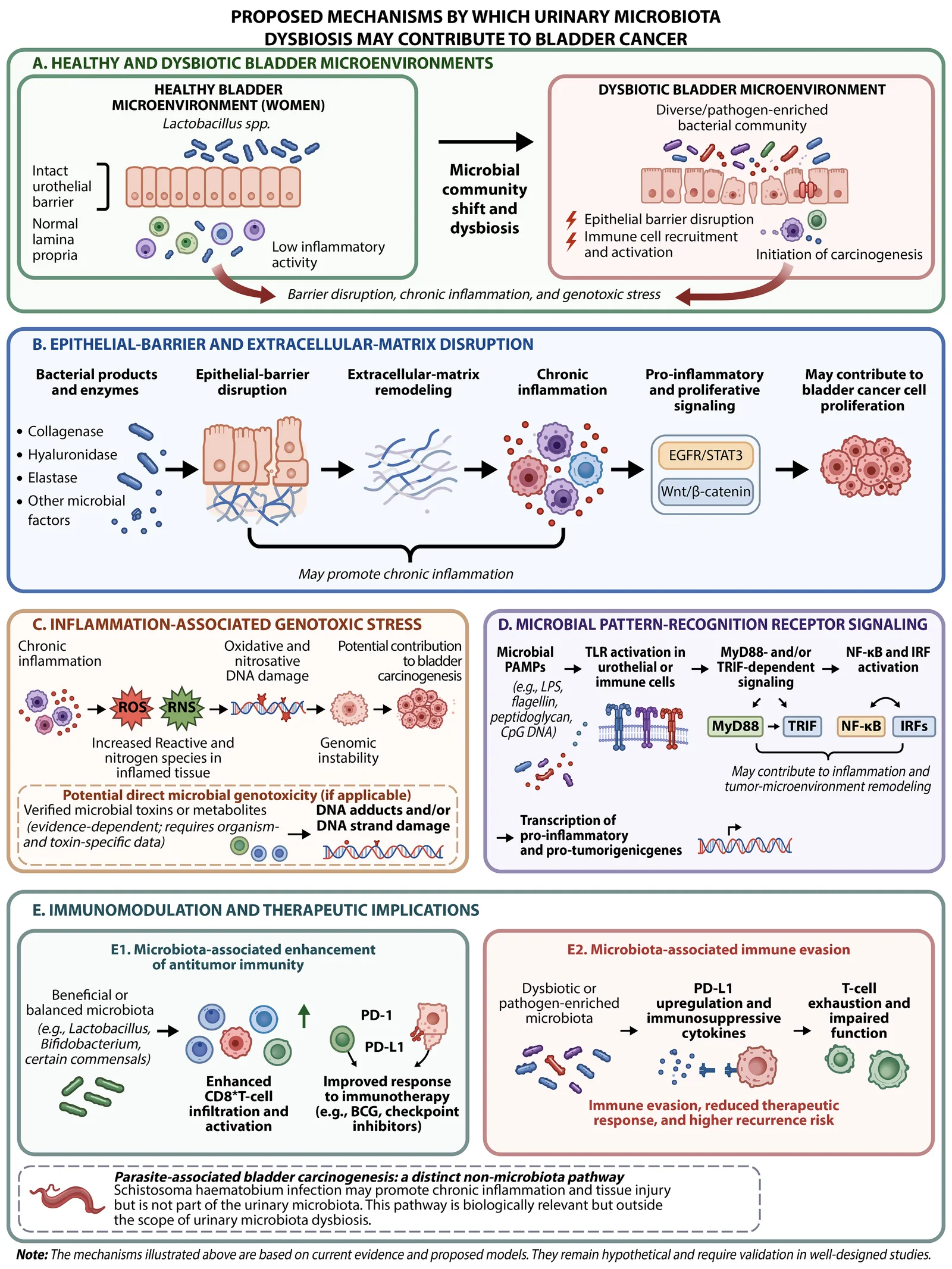
Proposed mechanisms by which urinary microbiota dysbiosis may contribute to bladder cancer. **(A)** Comparison of a healthy female bladder microenvironment, characterized by an intact urothelial barrier, low inflammatory activity, and a *Lactobacillus*-associated microbial profile, with a dysbiotic bladder microenvironment characterized by microbial community alteration, barrier disruption, and immune-cell activation. **(B)** Proposed effects of bacterial products and enzymes on epithelial-barrier integrity and extracellular-matrix remodeling, which may promote chronic inflammation and proliferative signaling. **(C)** Inflammation-associated genotoxic stress, in which increased production of reactive oxygen and nitrogen species may cause oxidative and nitrosative DNA damage, genomic instability, and contribute to bladder carcinogenesis. Potential direct microbial genotoxicity is shown as an evidence-dependent mechanism requiring organism- and toxin-specific validation. **(D)** Generalized microbial pattern-recognition receptor signaling, showing how microbial pathogen-associated molecular patterns may activate TLR-dependent MyD88 and/or TRIF pathways, followed by NF-κB and IRF activation and the expression of pro-inflammatory and pro-tumorigenic genes. **(E)** Potential microbiota-associated immunomodulatory effects, including enhancement of antitumor immunity or promotion of PD-L1 expression, T-cell exhaustion, immune evasion, and reduced therapeutic response.

The involvement of the microbiota in BC is multifaceted. Current evidence supports a potentially bidirectional and context-dependent relationship: some microorganisms or microbial products may contribute to tumor-promoting processes, whereas other taxa may represent secondary colonizers of an already altered tumor environment. Identifying and targeting key inflammatory pathways, bacterial agents, and immune modulators may offer new approaches for the prevention and treatment of BC. The distinct microbial communities and inflammatory environments in healthy versus pathological bladders are illustrated in **Figure 2**, highlighting the potential mechanistic role of dysbiosis in BC progression.

### 3.3 Correlation between microbiota and tumor stage and grade

Tumor stage and grade are critical determinants in the clinical management of BC, influencing treatment choices and prognostic outlooks. Studies have revealed distinct microbial profiles associated with different tumor stages and grades in BC patients (Parra-Grande et al., 2019). Hussein et al. (2021) found that *Cupriavidus* predominated in the urine of NMIBC patients, while *Haemophilus* and *Veillonella* were more common in MIBC patients. Other reports indicate significant enrichment of *Bacteroides* and *Faecalibacterium* in MIBC patients compared to NMIBC (Chipollini et al., 2020). However, some studies have reached conflicting conclusions. Mansour et al. (2020) found no significant differences in the urinary microbiome composition between NMIBC and MIBC, and reported no statistically significant differences in alpha and beta diversity between MIBC and NMIBC groups (Hussein et al., 2021; Oresta et al., 2021).

Additionally, Wu et al. (2018) found that *Herbaspirillum*, *Porphyrobacter*, and *Bacteroides* were highly expressed in patients at high risk for NMIBC recurrence and progression, suggesting that a higher bacterial abundance may serve as a potential biomarker for risk stratification and prognostic prediction. Furthermore, Oresta et al. (2021) reported an increased abundance of *Veillonella* and *Corynebacterium* as BC progressed, while Parra-Grande et al. (2022) noted a higher presence of *Enterococcus* in low-grade tumors. These findings highlight the complexity and heterogeneity of urinary microbiota across different stages and grades of BC, emphasizing the need for prospective studies to validate these microbial markers.

### 3.4 Application of urinary microbiota as diagnostic and prognostic biomarkers

Emerging evidence underscores the critical role of urinary microbiota as a potential biomarker for diagnosing and predicting the prognosis of BC, particularly in non-muscle-invasive BC (NMIBC). Vendrell et al. (2024) conducted a differential analysis identifying five genera—*Rhodanobacter*, *Cutibacterium*, *Alloscardovia*, *Moryella*, and *Anaeroglobus*—as significantly more abundant in BC patients compared to healthy controls. Among these, *Rhodanobacter* was detected in 20 cancer samples but completely absent in the controls. Conversely, 40 genera, including *Lactobacillus, Propionibacterium*, and *Bifidobacterium*, exhibited a marked decrease in cancer patients, suggesting that specific microbial taxa may serve as potential diagnostic biomarkers for BC. Further sequencing studies confirmed distinct microbial and metabolic changes in BC urine, and established a urinary microbiota-based diagnostic model with reliable AUC values (Tao et al., 2026).

The prognostic value of urinary microbiota has also been explored in relation to tumor recurrence (Herr and Donat, 2019). reported that patients with low-grade papillary NMIBC who exhibited asymptomatic bacteriuria had a lower recurrence rate, potentially due to local immune modulation triggered by bacteria. This finding suggests that urinary microbiota could serve as a predictor of tumor recurrence. Moreover, Zeng et al. (2020) demonstrated that NMIBC patients with recurrence exhibited higher alpha diversity in their urinary microbiota compared to those without recurrence. Specific genera, such as *Anoxybacillus* and *Micrococcus*, were enriched in the recurrence group, while the relative abundance of *Lactobacillus* was notably lower. These findings suggest that an increased abundance of certain taxa in the bladder may correlate with a higher risk of tumor recurrence. Similarly, Wu et al. (2018) highlighted that increased bacterial richness could serve as a predictive biomarker for NMIBC recurrence and progression.

**Figure 3:**
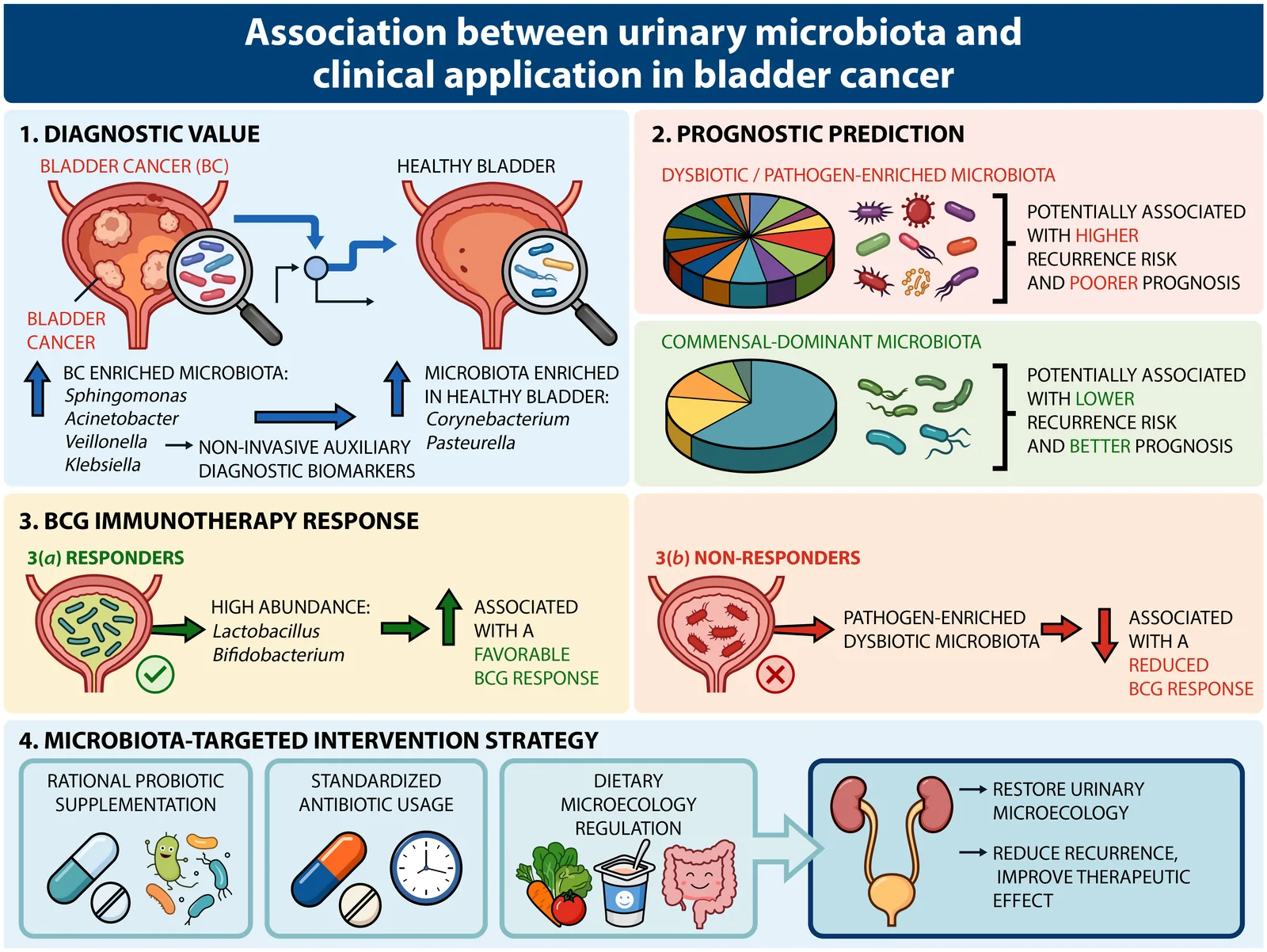
Association between urinary microbiota and clinical application in bladder cancer. The schematic summarizes four potential clinical applications of urinary microbiota profiling in bladder cancer. (1) Diagnostic value: taxa reported to differ between patients with bladder cancer and healthy controls may contribute to non-invasive classification. (2) Prognostic prediction: dysbiotic or pathogen-enriched microbial profiles may be associated with increased recurrence risk and poorer prognosis, whereas commensal-dominant profiles may be associated with more favorable outcomes. (3a–b) BCG immunotherapy response: microbial profiles reported in responders and non-responders are presented separately as potential indicators of treatment response. (4) Microbiota-targeted interventions: probiotics, standardized antibiotic use, and dietary regulation are proposed approaches for restoring urinary microbial homeostasis and potentially improving therapeutic outcomes. These associations and intervention strategies remain exploratory and require validation in larger prospective studies.

Collectively, these studies indicate that the diversity and richness of urinary microbiota may be associated with BC recurrence, emphasizing the importance of further research to identify specific microbial contributors and quantify their role in recurrence risk. Advancing our understanding of the urinary microbiota’s composition and its interplay with tumor biology could pave the way for novel diagnostic and prognostic tools, as well as potential therapeutic strategies targeting the microbiome. These multi-faceted clinical applications of urinary microbiota in BC are systematically summarized in **Figure 3**.

## 4 Clinical therapeutic value of targeted microbiota regulation

### 4.1 Correlation between urinary microbiota and BCG intravesical immunotherapy

Bacillus Calmette-Guérin (BCG) therapy, an intravesical instillation of an attenuated strain of *Mycobacterium bovis*, is the gold standard adjuvant treatment for high-risk NMIBC, achieving an initial complete response rate of 55-65% in high-risk tumors and 70-75% in carcinoma in situ (Pettenati and Ingersoll, 2018). Although BCG’s anti-cancer mechanism is not fully understood, it is thought to work by inducing an anti-tumor immune response within the bladder (Heidar et al., 2023). Recent evidence has highlighted specific metabolic and genetic alterations associated with successful BCG responses (Knorr et al., 2024). Notably, two metabolic pathways, assimilatory sulfate reduction and the reductive citrate cycle, were significantly enriched in BCG responders. Furthermore, certain genes exhibited marked changes in expression. For example, indole-3-pyruvate decarboxylase, a gene involved in tryptophan metabolism, showed a dramatic log2 fold change of 23.40 (*P* < 0.001), suggesting a potential role in modulating the tumor microenvironment or immune response. Similarly, β-lactamase, with a log2 fold change of 4.23 (*P* = 0.035), may influence bacterial persistence or immune activation within the bladder. These findings imply that specific metabolic processes and gene expressions could play a crucial role in determining the therapeutic efficacy of BCG treatment.

The relationship between urinary microbiota and the response to BCG therapy remains a topic of ongoing debate (Lloyd, 2024). Emerging evidence highlights differences in microbial composition, richness, and diversity between BCG responders and non-responders. For instance, *Lactobacillus* has been found to be significantly enriched in BCG responders, suggesting its potential role in modulating the bladder’s immune environment to enhance therapeutic efficacy (Knorr et al., 2024). Similarly, *Bifidobacterium* was frequently detected in responders and has been associated with prolonged recurrence-free survival (Min et al., 2024), pointing to the possible beneficial effects of these commensal bacteria in improving BCG outcomes. A systematic literature review also confirmed that Lactobacillus is the most dominant genus in BCG responders, while Streptococcus is highly enriched in BCG non-responders (Jian et al., 2025). Notably, the post-BCG group showed increased Pseudomonas, Lactococcus and Bacillus, along with enhanced microbial metabolic pathways including amino acid, lipopolysaccharide and glucose degradation (Li et al., 2026).

Other studies have identified distinct microbial profiles associated with BCG responsiveness. Boban et al. (2025) reported reduced microbial diversity during BCG therapy and found that a higher pretreatment abundance of *Aureispira* and lower levels of *Negativicoccus succinivorans* were characteristic of BCG non-responders. Hussein et al. (2021) reported higher levels of genera such as *Serratia*, *Brochothrix*, *Negativicoccus*, *Escherichia-Shigella*, and *Pseudomonas* in patients who responded well to BCG therapy. These bacteria may influence the local immune response or contribute to creating a bladder microenvironment favorable for BCG activity.

Contrastingly, a study by Heidrich et al. (2024) suggested that the bladder microbiota does not undergo significant alterations following intravesical BCG therapy, raising questions about whether pre-existing microbial compositions are more critical in determining BCG efficacy than therapy-induced changes.

Collectively, these findings suggest that specific microbial communities might play a role in modulating the effectiveness of BCG treatment. However, the variability in microbial profiles identified across studies—such as the presence of Lactobacillus, Bifidobacterium, or other genera—indicates that no single microbial signature has been consistently associated with BCG response. Further research is needed to elucidate whether these microbes act through shared functional pathways, such as immune activation or metabolic modulation, to influence treatment outcomes. Organizing future discussions based on microbial functions or similarities could clarify these complex interactions and advance our understanding of the microbiota-BCG relationship (**Table 2**).

**Table 2:** Studies reporting microbiota-associated features related to BCG response in BC.

References	Sample size (BC)	Microbiota technique	Sample	Microbiota related to BCG response
Knorr et al. (2021)	26 NMIBC patients: 14 responders and 12 non-responders	16sRNA	Tissue	*Corynebacterium ↑*
				*Pseudomonas ↑*
Knorr et al. (2022)	47 NMIBC patients: 23 responders and 24 non-responders	16sRNA	Tissue	*Lactobacillus ↑*
				*Corynebacterium ↓*
Hussein et al. (2021)	43 BC patients; BCG-response analysis was limited to 11 BCG-treated NMIBC patients	16sRNA	Urine	*Serraia ↑*
				*Brochothrix ↑*
				*Negativicoccus ↑*
				*Escherichia-Shigella Pseudomonas↑*
Heidrich et al. (2024)	32 NMIBC patients undergoing BCG therapy; 41 non-oncological controls were also included	16sRNA	Urine	*NC
Min et al. (2024)	58 BC patients undergoing BCG therapy; 29 benign controls were also included	16sRNA	Urine	*Bifidobacterium ↑*
Knorr et al. (2024)	47 archival FFPE tumor samples; 10 fresh tumor samples and 11 urine samples were also prospectively collected	16S rRNA and shotgun metagenomics	Tissue and urine	*Lactobacillus ↑*

*NC, No change. ↑, significantly increased; ↓, significantly decreased.

### 4.2 Probiotic-based and other microbiota-modulating strategies

Antibiotics, frequently used alongside cancer treatments, can significantly impact urinary microbiota composition. A study on urogenital microbiota found that antibiotic exposure reduced *Lactobacillus* and *Finegoldia* abundance, while *Escherichia coli* levels increased (Mulder et al., 2019). Moreover, evidence suggests that antibiotic use during neoadjuvant pembrolizumab immunotherapy in MIBC patients correlates negatively with recurrence-free survival and pathologic complete response (Huang et al., 2019; Tinsley et al., 2020).

A single study (Ginwala et al., 2025) demonstrated that *Granulicatella* and *Proteus* were enriched in neoadjuvant chemotherapy non-responders, while *Enterococcus faecalis* was abundant in responders; microbial profiles derived from urine and paired bladder tumor tissue showed approximately 32% overlap, single-dose perioperative antibiotics caused slight transient microbial changes, and urine-derived Gammaproteobacteria could attenuate gemcitabine efficacy.

Probiotics, particularly lactobacilli, have shown promise in reducing NMIBC recurrence (Dadgar-Zankbar et al., 2024). For instance, oral administration of *Lactobacillus* preparations has shown protective effects against postoperative recurrence of BC, potentially implicating the microbiome in recurrence dynamics (James et al., 2023). Similarly, fermented dairy products, which contain *Lactobacillus*, have been associated with a reduced risk of BC (Zhang et al., 2018). However, further research is needed to confirm the protective effects of probiotics against BC. Additionally, whether modulating microbiota through probiotics, antibiotics, or other therapeutic approaches could aid in BC prevention and treatment remains an area of active investigation. Emerging evidence highlights the potential of microbiota-targeted interventions, such as probiotic supplementation or microbiome-based therapies, as promising adjunct strategies to enhance BC treatment outcomes.

## 5 Conclusion and future directions

Changes in the text: Current evidence indicates that microbial communities can be detected in urine and bladder tissue and that their composition may vary according to sex, specimen-collection method, tumor stage, recurrence status, and treatment exposure. However, no reproducible BC-specific microbial signature has yet been established, and most reported associations remain observational. Marked heterogeneity among studies—including small cohorts, the susceptibility of low-biomass samples to contamination, inconsistent collection and storage methods, variation in 16S rRNA target regions and bioinformatic pipelines, and incomplete adjustment for antibiotics, smoking, age, sex, comorbidities, and tumor characteristics—limits causal interpretation and precludes immediate clinical application.

Future research should prioritize standardized, multicenter, prospective studies with prespecified protocols for specimen collection, storage, contamination control, DNA extraction, sequencing, and reporting. Voided urine, catheterized urine, and bladder tissue should be analyzed as distinct specimen types. Whenever feasible, paired urine–tissue sampling and longitudinal collection before and after transurethral resection, BCG therapy, chemotherapy, or immunotherapy should be incorporated. Beyond 16S rRNA profiling, shotgun metagenomics, metatranscriptomics, metabolomics, and spatial approaches are needed to provide strain- and function-level resolution and to distinguish viable resident microorganisms from contaminants or transient microbial DNA. Mechanistic studies using bladder organoids, urothelial co-culture systems, and relevant *in vivo* models should further determine whether candidate microorganisms or metabolites directly alter barrier integrity, inflammatory signaling, genomic stability, antitumor immunity, or drug metabolism.

For clinical translation, microbiota-based diagnostic and prognostic models must be externally validated against established tools such as cystoscopy, urine cytology, and clinicopathological risk models. Candidate markers of recurrence, progression, and BCG response require adequately powered validation cohorts with clinically meaningful endpoints. Microbiota-modulating interventions, including probiotics, antibiotics, dietary approaches, and targeted microbial products, should not be adopted empirically, because nonspecific manipulation may eliminate beneficial communities, select resistant organisms, or reduce the efficacy of anticancer therapy. Thus, urine- and bladder tissue-associated microbiota represent promising sources of biomarkers and biological hypotheses, but methodological standardization, causal validation, and prospective clinical testing are essential before microbiota-guided management can enter routine BC care.
